# Fatality Rate and Survival Time of Laboratory-Confirmed COVID-19 for Patients in England During the First Wave of SARS-CoV-2 Infection: A Modelling Study

**DOI:** 10.7759/cureus.16899

**Published:** 2021-08-05

**Authors:** Christopher R Hillyar, Anjan Nibber, Christine E Jones, Mark G Jones

**Affiliations:** 1 John Radcliffe Hospital, Oxford University Hospitals NHS Foundation Trust, Oxford, GBR; 2 Clinical and Experimental Sciences, University Hospital Southampton NHS Foundation Trust, Southampton, GBR

**Keywords:** covid-19, sars-cov-2, fatality rate, mortality rate, survival time, modelling, joinpoint

## Abstract

Background

Fatality rate estimates for coronavirus disease 2019 (COVID-19) have varied widely. A major confounding factor in fatality rate estimates is the survival time (time from diagnosis to death). Predictive models that incorporate the survival time benefit from greater accuracy due to the elimination of sampling bias. This study outlines a survival time-based predictive model that estimates a precise fatality rate for patients with laboratory-confirmed COVID-19. This model was utilised to predict deaths for COVID-19 patients who died during the first wave of severe acute respiratory syndrome coronavirus 2 (SARS-CoV-2) infection in England.

Methodology

This study included Public Health England (PHE) data for cumulative laboratory-confirmed COVID-19 cases (n = 143,463) and deaths (n = 30,028) that were reported by PHE between 30 January and 14 May 2020 in England, that is, from the first COVID-19 case in England and the most recently available data at the time of conducting this study. Fatality rate and survival time were estimated by linear regression analysis. This enabled the predicted cumulative COVID-19 deaths to be calculated up to 21 May 2020. Time periods with significantly different rates in daily deaths were identified using Joinpoint trend analysis.

Results

A fatality rate of 21.9% (95% confidence interval = 21.8% to 22.0%) with a survival time of seven days was determined for patients in England with laboratory-confirmed COVID-19 during the first wave of SARS-CoV-2 infection. Based on these estimates, predicted trends for cumulative and daily laboratory-confirmed COVID-19 deaths were generated with >99% and >96% accuracy with reported data, respectively. This model predicted that the number of cumulative laboratory-confirmed COVID-19 deaths in England was likely to be 31,420 by 21 May 2020. Joinpoint trend analysis identified significant differences in predicted daily laboratory-confirmed COVID-19 deaths during the following periods: 10.5 (6 to 17 March), 111.0 (17 to 27 March), 446.8 (27 March to 4 April), 817.0 (4 to 23 April), 536.3 (23 April to 7 May), and 266.7 (7 to 21 May) daily deaths (P < 0.001).

Conclusions

During the first wave of SARS-CoV-2 infection in England, the fatality rate of laboratory-confirmed COVID-19 was 21.9%. The survival time of these patients was seven days. The predictive model presented in this study can be adapted for estimating COVID-19 deaths in different geographical regions. As such, this study has utility for clinicians, scientists, and policymakers responding to new waves of SARS-CoV-2 infection because the methodology can be applied to more recent time periods as new data for COVID-19 cases and deaths become available.

## Introduction

In December 2019, the global pandemic of coronavirus disease 2019 (COVID-19) started due to an outbreak in Wuhan, Hubei Province, China, of severe acute respiratory syndrome coronavirus 2 (SARS-CoV-2) [1]. The SARS-CoV-2 virus causes a range of symptoms, including fever, cough, chest tightness, dyspnoea, fatigue, and myalgia, that may ultimately place patients at risk of ventilatory support in intensive care and death [2]. During the early stages of the pandemic, fatality rate estimates from different geographical regions throughout the world for COVID-19 patients ranged from 0.7% to 33.3% [3-9]. The majority of epidemiological studies based fatality rate estimates on cumulative cases and deaths, without accounting for the time from laboratory diagnosis to the time of death (survival time). A systematic review concluded that this approach suffers from severe sampling bias due to the inclusion of patients who have neither died nor recovered from COVID-19 [10]. The incorporation of the survival time into models for estimating the fatality rate can reduce this form of bias and provide more accurate estimates. Russel et al. reported a mean survival time of 13 days for COVID-19 patients from the Diamond Princess cruise ship who died from SARS-CoV-2 infection [11]. An epidemiological study by Baud et al. excluded patients who had not yet had an outcome from COVID-19 by incorporating a survival time of 14 days into fatality rate estimates inside and outside of China (5.6% and 15.2%, respectively) [4].

In the United Kingdom, COVID-19 testing during the first wave of SARS-CoV-2 infection mainly focused on the National Health Service (NHS) and Public Health England (PHE) laboratory swab testing of people with clinical need, as well as testing of critical key workers in the NHS [12]. In England, PHE has published data for cumulative cases and deaths for patients in hospitals and the community with laboratory-confirmed COVID-19 [13]. This study aimed to estimate the fatality rate for patients in England with laboratory-confirmed COVID-19 by basing this estimate on the actual survival time of patients who died from laboratory-confirmed SARS-CoV-2 infection. It was hypothesised that a survival time-based predictive model that produced precise estimates for predicted COVID-19 deaths compared to reported COVID-19 deaths would indicate the actual survival time of patients with laboratory-confirmed COVID-19 who have died from SARS-CoV-2 infection. This model could then be utilised to estimate predicted cumulative laboratory-confirmed COVID-19 deaths over time and to analyse trends in predicted daily deaths. This study was previously presented as a meeting abstract at the 2021 British Thoracic Society Winter Meeting on February 17, 2021 [14].

## Materials and methods

This study has been reported as per the Transparent Reporting of a Multivariable Prediction Model for Individual Prognosis or diagnosis guidelines.

Dataset and definitions

Cumulative laboratory-confirmed COVID-19 cases and deaths published by PHE between 30 January and 14 May 2020 (the study period) were accessed on 21 May 2020 and analysed under an Open Government Licence without the requirement for ethical approval [13]. Only data for England were included. Laboratory-confirmed COVID-19 cases were defined as people who received a positive test result for COVID-19 infection, with the specimen date serving as the date of diagnosis [12]. This included laboratory-confirmed reports from the ‘Pillar 1’ testing programme, that is, NHS and PHE laboratory swab testing by reverse transcription-polymerase chain reaction (RT-PCR) of people with clinical need and critical key workers in the NHS [12]. Laboratory-confirmed COVID-19 deaths were defined as all deaths reported to PHE with a laboratory-confirmed report of COVID-19 (including at post-mortem) in any setting [15]. Data handling was performed using Microsoft Excel.

COVID-19 fatality rate and survival time

To estimate an accurate fatality rate for patients in England with laboratory-confirmed COVID-19, a model was developed to quantify the precision of estimates of predicted deaths attributable to laboratory-confirmed COVID-19. These estimates were generated from predictive models that incorporated a range of different survival times (0 to 16 days). Survival time was defined as the time from diagnosis (date of laboratory specimen) to the time of death (reported by PHE). The actual survival time for laboratory-confirmed COVID-19 cases (where the outcome was death) corresponded to the specific survival time incorporated in the predictive model, which generated the most precise estimates for predicted laboratory-confirmed COVID-19 deaths. The fatality rate for a given survival time (that is, the fatality rate that would be expected if a patient survived for a period of time equivalent to the survival time) was first estimated by plotting the reported cumulative laboratory-confirmed COVID-19 deaths (on day *d*) against the reported cumulative laboratory-confirmed COVID-19 cases (on day *d* minus the survival time *n*). Linear regression was utilised to quantify the relationship between these two variables. The slope of the regression curve corresponded to the 1/fatality rate.

Predicted COVID-19 deaths and sensitivity analysis

After calculating the fatality rates generated by each predictive model, the fatality rates were utilised to produce a range of estimates for predicted cumulative laboratory-confirmed COVID-19 deaths (on day *d* plus survival time *n*) by dividing the reported cumulative laboratory-confirmed COVID-19 cases (on day *d*) by the fatality rate from the predictive model with the corresponding survival time. Linear regression was utilised to quantify the precision of the estimates for the predicted cumulative laboratory-confirmed COVID-19 deaths (on day *d* plus survival time *n*) by plotting this variable against the corresponding cumulative laboratory-confirmed COVID-19 deaths reported on the same day. The standard errors from these regression curves were utilised to derive the precision (1/standard error) and goodness-of-fit (R^2^) of each set of estimates for predicted cumulative laboratory-confirmed COVID-19 deaths (on day *d* plus survival time *n*). The precision of each set of estimates was plotted against the survival time incorporated by the corresponding predictive model to identify which predictive model and survival time generated the best estimates for predictive cumulative laboratory-confirmed COVID-19 deaths (on day *d* plus survival time *n*). Thus, the specific survival time for patients in England who died from laboratory-confirmed COVID-19 during the study period was derived from the ability of a specific survival time-based predictive model to generate precise estimates of predicted cumulative laboratory-confirmed COVID-19 deaths. Statistical analysis was performed using GraphPad Prism 8.

Similarity between trends for predicted and reported COVID-19 deaths

Based on the survival time (*n_s_*) incorporated by the predictive model that produced the best estimates for predicted cumulative laboratory-confirmed COVID-19 deaths, predicted cumulative laboratory-confirmed COVID-19 deaths (on day *d* plus *n_s_* days) were plotted over time. Predicted cumulative laboratory-confirmed COVID-19 deaths (on day *d* plus *n_s_* days) were calculated by multiplying the reported cumulative laboratory-confirmed COVID-19 cases (on day *d*) by the fatality rate from the predictive model that incorporated a survival time of *n_s_* days. Predicted daily laboratory-confirmed COVID-19 deaths (on day *d* plus *n_s_* days), which were calculated from predicted cumulative laboratory-confirmed COVID-19 deaths (on day *d* plus *n_s_* days), were also plotted over time. The percentage difference between predicted and reported trends (for both cumulative and daily COVID-19 deaths) was calculated by dividing the Euclidean distance (i.e. the square root of the sum of the squared differences between the predicted and reported trends on each day *d* over time) by the area under the curve for the trend of reported (cumulative or daily) deaths. The percentage similarity, or the accuracy, of the trends generated by the predictive model was equal to 100 minus the percentage difference.

Joinpoint trend analysis

Joinpoint trend analysis was utilised to assess significant changes in trends for predicted and reported cumulative laboratory-confirmed COVID-19 deaths (on day *d* plus *n_s_* days). This generated a series of linear regression curves interrupted by ‘joinpoints’, between which the mean daily deaths were significantly different. Thus, time periods were identified during which the mean daily laboratory-confirmed COVID-19 deaths (on day *d* plus *n_s_* days) significantly increased or decreased. Joinpoint trend analysis was conducted using Joinpoint Statistical Software 4.7.0.0, available from the Division of Cancer Control and Population Science, National Institutes of Health [16].

## Results

Estimates for fatality rate and survival time

**Figure 1:**
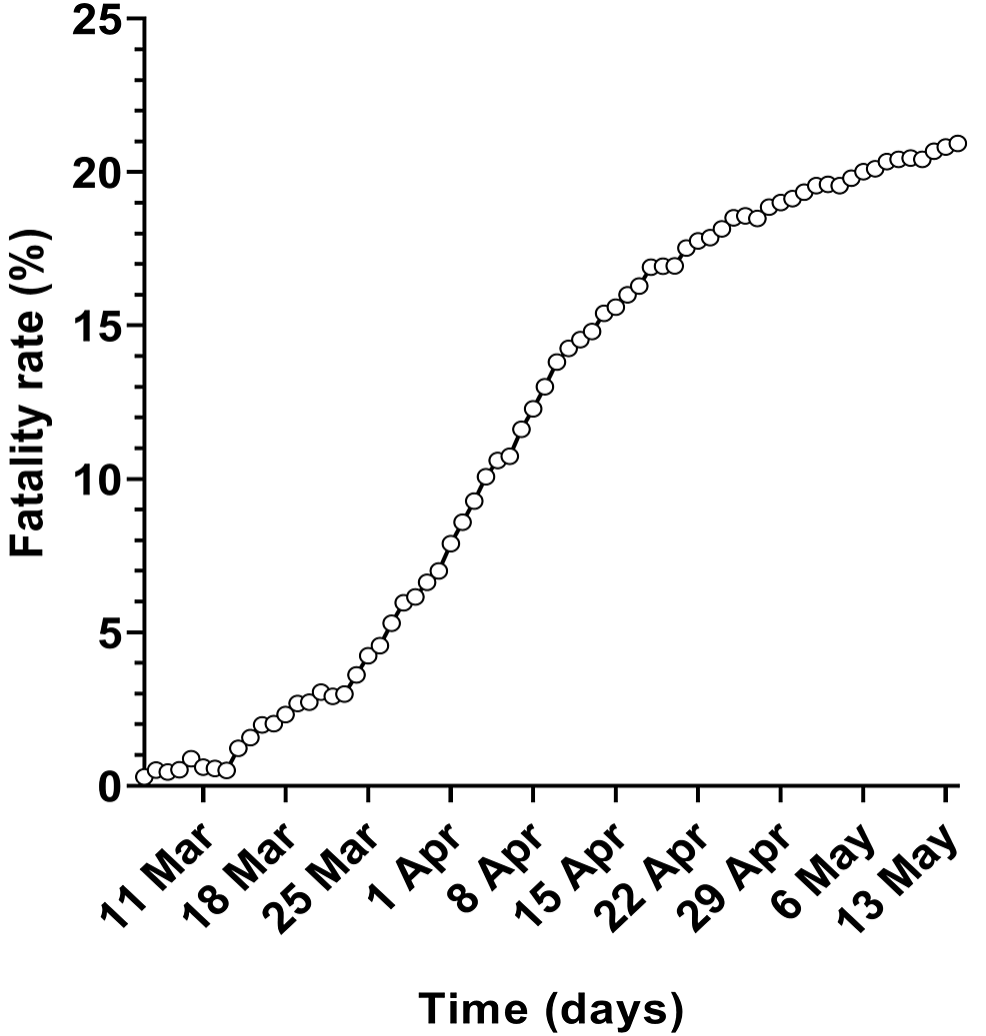
COVID-19 daily fatality rate during the first wave of SARS-CoV-2 infection in England between 30 January and 14 May 2020. The daily fatality rate was calculated from the ratio of reported cumulative laboratory-confirmed COVID-19 deaths (on day *d*) to the cumulative laboratory-confirmed COVID-19 cases reported on the same day. COVID-19: coronavirus disease 2019; SARS-CoV-2: severe acute respiratory syndrome coronavirus 2

This study included data for 143,463 cases and 30,028 deaths related to laboratory-confirmed COVID-19 in England reported between 30 January and 14 May 2020. Daily fatality rates, which were calculated by dividing cumulative laboratory-confirmed COVID-19 deaths on day *d* by the cumulative cases on the same day, ranged from 0.3% to 20.9% (Figure 1).

**Figure 2:**
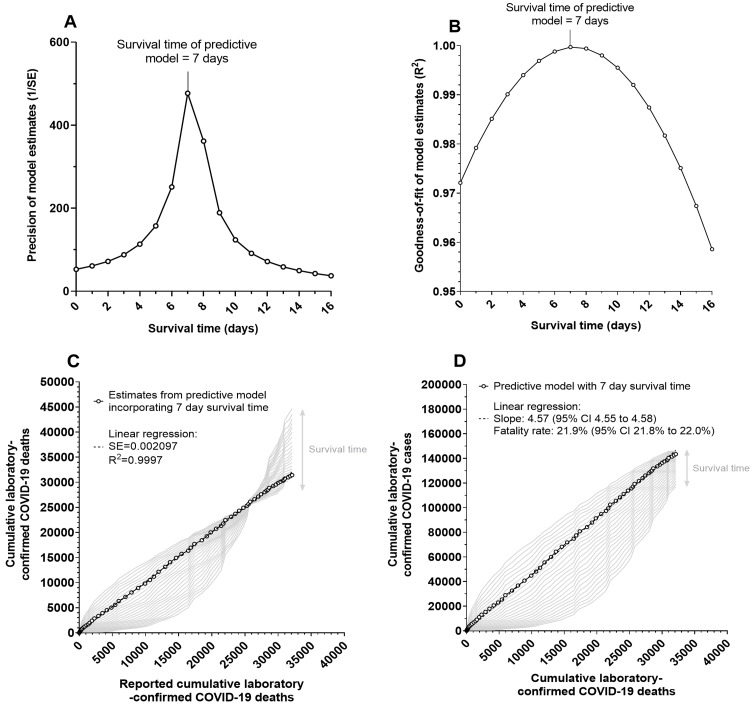
Survival time and fatality rate for patients in England with laboratory-confirmed COVID-19 who died from SARS-CoV-2 infection in England between 30 January and 14 May 2020. (A) Precision (1/SE) and (B) goodness-of-fit (R^2^) of estimates for predicted cumulative laboratory-confirmed COVID-19 deaths (on day *d* plus survival time *n*) plotted against the corresponding survival time incorporated by the predictive model from which the estimates were generated. (C) Linear regression analysis of estimates for predicted cumulative laboratory-confirmed COVID-19 deaths (on day *d* plus survival time *n*) plotted against the actual cumulative laboratory-confirmed COVID-19 deaths reported on the same day (linear regression of estimates from the seven-day predictive model: precision (1/SE) = 476.9; SE = 0.002097; R^2^ = 0.9997). (D) Linear regression analysis of reported cumulative laboratory-confirmed COVID-19 deaths (reported on day *d*) plotted against cumulative laboratory-confirmed COVID-19 cases (reported on day *d* minus survival time *n*; linear regression for the seven-day predictive model: slope = 4.57 (95% CI: 4.55 to 4.58); fatality rate (1/slope) = 21.9% (95%: CI 21.8% to 22.0%). CI: confidence interval; COVID-19: coronavirus disease 2019; SARS-CoV-2: severe acute respiratory syndrome coronavirus 2; SE: standard error from linear regression curve used to estimate the precision of the predictions against reported data

Predictive models that incorporated a range of survival times (0 to 16 days) were utilised to produce a range of estimates for predicted cumulative laboratory-confirmed COVID-19 deaths. These estimates varied in precision when assessed through linear regression analysis against actual cumulative laboratory-confirmed COVID-19 deaths reported on the same day. Precision was calculated from the reciprocal of the standard error (SE) of the regression curve. The predictive model based on a seven-day survival time produced the most precise, best-fitting estimates for predicted cumulative laboratory-confirmed COVID-19 deaths (precision (1/SE) = 476.9; R^2^ = 0.9997) (Figures 2A-2C). This suggested that for patients with laboratory-confirmed COVID-19 in England who died during the first wave of SARS-CoV-2 infection in hospital or in the community, the time from laboratory diagnosis to death was seven days. The estimates for predicted cumulative laboratory-confirmed COVID-19 deaths that were produced by the predictive model were generated from the calculated fatality rate. The fatality rate was calculated by plotting the reported cumulative laboratory-confirmed COVID-19 deaths (on day *d*) against the cumulative laboratory-confirmed COVID-19 cases (on day *d* minus seven days) reported from 6 March to 14 May 2020. Linear regression analysis confirmed that the fatality rate for patients who died from laboratory-confirmed COVID-19 during the first wave of SARS-CoV-2 infection in England was 21.9% (95% confidence interval (CI) = 21.8% to 22.0%) (Figure 2D).

Accuracy of the predictive model and changes in trends in daily deaths

**Figure 3:**
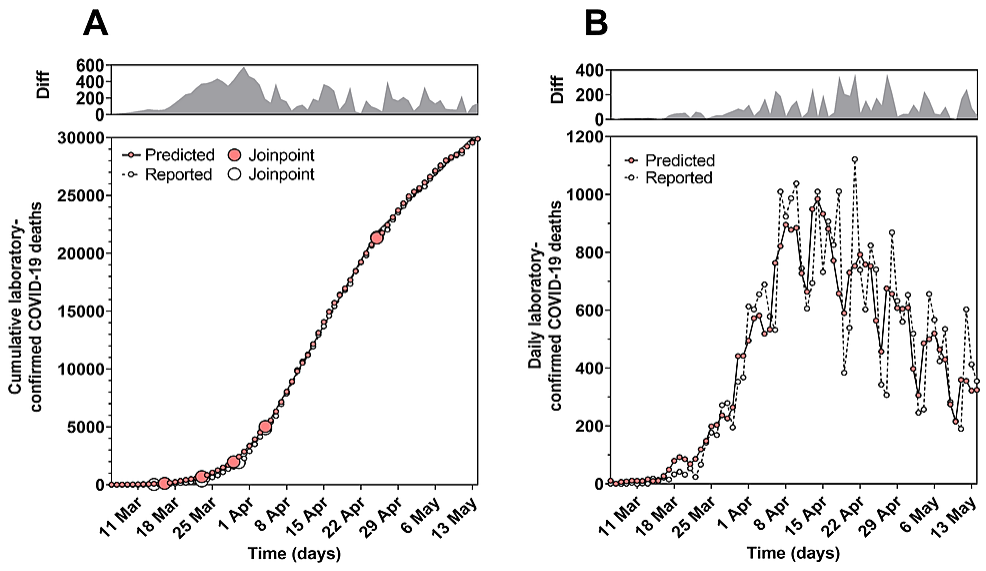
Comparison of predicted and reported trends in cumulative and daily laboratory-confirmed COVID-19 deaths during the first wave of SARS-CoV-2 infection in England between 30 January and 14 May 2020. (A) Joinpoint trend analysis of trends for predicted and reported cumulative laboratory-confirmed COVID-19 deaths plotted from 6 March to 14 May 2020. (B) Trends for predicted and reported daily laboratory-confirmed COVID-19 deaths plotted from 6 March to 14 May 2020. Top panels show the difference between the predicted and reported trends. COVID-19: coronavirus disease 2019; Diff: difference; SARS-CoV-2: severe acute respiratory syndrome coronavirus 2

Utilising the predictive model, estimates for predicted cumulative laboratory-confirmed COVID-19 deaths (on day *d* plus seven days) were plotted over time (from 6 March to 14 May 2020). Cumulative laboratory-confirmed COVID-19 deaths (reported on the same day) were also plotted over the same time period (Figure 3A). The percentage difference between the predicted and reported trends in cumulative laboratory-confirmed COVID-19 deaths was 0.3%. Joinpoint trend analysis of the trends in predicted cumulative laboratory-confirmed COVID-19 deaths identified time intervals during which the mean daily laboratory-confirmed COVID-19 deaths significantly increased or decreased. The Monte Carlo permutation method fitted five joinpoints to the time series (P < 0.001). Predicted daily laboratory-confirmed COVID-19 deaths were significantly different between these joinpoints (Table 1). Joinpoint trend analysis demonstrated a similar result for trends in actual cumulative laboratory-confirmed COVID-19 deaths reported on the same day. This suggested daily deaths in patients in England with laboratory-confirmed COVID-19 initially increased in mid-March to early April, and then subsequently reduced in late April. Predicted and reported daily laboratory-confirmed COVID-19 deaths (plotted from 6 March to 14 May 2020) also demonstrated similar trends over time (Figure 3B). The percentage difference between the predicted and reported trends in daily laboratory-confirmed COVID-19 deaths was 3.6%. Together, these findings confirm the utility of the predictive model, which predicted trends for cumulative and daily laboratory-confirmed COVID-19 deaths with an accuracy of 99.7% and 96.4%, respectively.

**Table 1:** Joinpoint trend analysis of predicted and reported COVID-19 deaths during the first wave of SARS-CoV-2 infection in England between 30 January and 14 May 2020. Joinpoint trend analysis of segmented trends in predicted and reported daily laboratory-confirmed COVID-19 deaths (from 6 March to 14 May 2020). †The optimal number of joinpoints was determined by a test of significance based on a Monte Carlo permutation method. *Segmented trends between joinpoints were tested to determine if they were significantly different from no change (0). COVID-19: coronavirus disease 2019; SARS-CoV-2: severe acute respiratory syndrome coronavirus 2

Trends in laboratory-confirmed COVID-19 deaths					
Predicted			Reported		
Segmented trends (P < 0.001)†	Rate of daily deaths	P-value*	Segmented trends (P < 0.001)†	Rate of daily deaths	P-value*
6 to 16 March	9.7	<0.001	6 to 14 March	1.4	0.0186
16 to 23 March	80.6	<0.001	14 to 23 March	29.1	<0.001
23 to 29 March	209.2	<0.001	23 to 30 March	211.8	<0.001
29 March to 4 April	496.7	<0.001	30 March to 4 April	591.1	<0.001
4 to 16 April	805.7	<0.001	4 to 16 April	808.8	<0.001
25 April to 14 May	453.6	<0.001	25 April to 14 May	456.6	<0.001

Joinpoint trend analysis of predicted cumulative COVID-19 deaths

**Figure 4:**
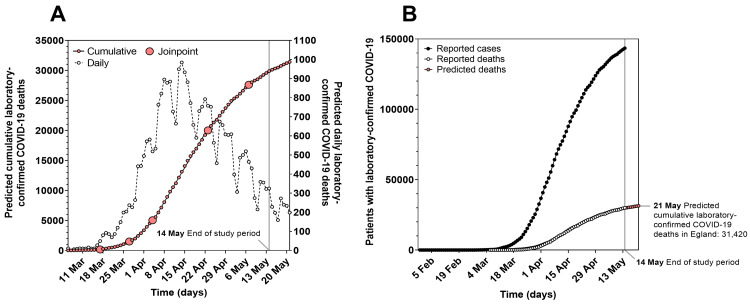
Predicted laboratory-confirmed COVID-19 deaths in England from 30 January to 21 May 2020. (A) Joinpoint trend analysis of trends for predicted cumulative and daily laboratory-confirmed COVID-19 deaths (from 6 March to 21 May 2020). (B) Predicted cumulative laboratory-confirmed COVID-19 deaths (from 30 January to 21 May 2020) plotted alongside the reported cumulative laboratory-confirmed COVID-19 cases and deaths (from 30 January to 14 May 2020). COVID-19: coronavirus disease 2019

Based on the fatality rate of 21.9%, the predictive model was utilised to extend predicted cumulative laboratory-confirmed COVID-19 deaths in England beyond the end of the study period to 21 May 2020. Joinpoint trend analysis identified time intervals during which the predicted daily laboratory-confirmed COVID-19 deaths significantly increased and decreased (Figure 4A). The Monte Carlo permutation method fitted five joinpoints to the time series (P < 0.001). Daily deaths were again significantly different between these joinpoints (Table 2). Predicted daily laboratory-confirmed COVID-19 deaths, plotted from 6 March to 21 May 2020, demonstrated an initial increase in mid-March to early April, followed by a reduction in early May. Predicted cumulative laboratory-confirmed COVID-19 deaths (from 30 January to 21 May 2020) were plotted alongside the reported cumulative laboratory-confirmed COVID-19 cases and deaths (from 30 January to 14 May 2020) (Figure 4B), highlighting that 31,420 predicted cumulative laboratory-confirmed COVID-19 deaths occurred in England by 21 May 2020.

**Table 2:** Joinpoint trend analysis of trends in predicted laboratory-confirmed COVID-19 deaths in England from 30 January to 21 May 2020. Joinpoint trend analysis of segmented trends in predicted daily laboratory-confirmed COVID-19 deaths (from 6 March to 21 May 2020). †The optimal number of joinpoints was determined by a test of significance based on a Monte Carlo permutation method. *Segmented trends between joinpoints were tested to determine if they were significantly different from no change (0). COVID-19: coronavirus disease 2019

Trends in predicted laboratory-confirmed COVID-19 deaths		
Segmented trends between joinpoints (P < 0.001)†	Rate of daily deaths	P-value*
6 to 17 March	10.5	<0.001
17 to 27 March	111.0	<0.001
27 March to 4 April	446.8	<0.001
4 to 23 April	817.0	<0.001
23 April to 7 May	536.3	<0.001
7 to 21 May	266.7	<0.001

## Discussion

This study provides precise estimates for the fatality rate and survival time for patients who died from laboratory-confirmed COVID-19 during the first wave of SARS-CoV-2 infection in England. The fatality rate for patients with laboratory-confirmed COVID-19 in England between 30 January and 14 May 2020 corresponded to approximately one-fifth of patients, with four-fifths predicted to have made a full recovery. During the first wave of SARS-CoV-2 infection in England, patients with a clinical need for testing who died from COVID-19 were likely to do so after seven days from diagnosis based on the date of the specimen obtained for diagnostic laboratory testing. The clinical relevance of these findings includes (a) an estimation of the risk of death for patients who are admitted to hospitals in England with COVID-19 (21.9%) and (b) an estimation of the likely survival time of patients who die from COVID-19 in hospitals in England. Although these findings were based on PHE data from a specific time period (30 January to 14 May 2020) which may no longer reflect current testing and treatment practices and do not account for age-specific differences in fatality rate and survival time, these findings are important considerations as a general guide to inform clinical management of, and communication with, patients (and their families) who have a clinical need for hospital admission. These findings, therefore, add to our understanding of the probable course of symptomatic (and potentially more severe) COVID-19 cases.

The predictive model reported in this study provides a simple method that can be adapted and based on data from other geographic areas for estimating the fatality rate of patients with laboratory-confirmed COVID-19, where testing is performed for clinical need. For example, for any country or region for which data for cumulative cases and deaths are available, the method outlined in this study can be applied to determine the fatality rate and survival time in that specific geographic area over any given time period. The predicted trends for cumulative and daily laboratory-confirmed COVID-19 deaths had an accuracy of >99% and >96%, respectively. Joinpoint trend analysis of the estimates from this model identified time periods during which significant increases and decreases in daily laboratory-confirmed COVID-19 deaths are likely to have occurred. The findings from the Joinpoint trend analysis were similar for reported COVID-19 deaths. The predictive model was utilised to extend predictive cumulative laboratory-confirmed COVID-19 deaths to 21 May 2020, when 31,420 cumulative deaths were predicted to have occurred.

Strengths and weaknesses of this study

In England, as the prioritisation of testing during the first wave of SARS-CoV-2 infection was for patients with clinical need, individuals with symptomatic but mild disease in the community as well as asymptomatic individuals, which have been estimated to comprise 18% of infected individuals [17], were not included in this study. Therefore while these findings are generalisable to patients with clinical need (typically those hospitalised or care home residents), it precludes the application of the results to the general population and suggests that the fatality rate for all individuals in the wider population who become infected with SARS-CoV-2 is much lower.

The false-negative rate of RT-PCR at detecting SARS-CoV-2 has been reported to range from 0% to as high as 21% [18-20] and may even be higher through thermal inactivation of specimens [21]. These issues may affect the estimates of fatality rate presented in this study, while strategies for collecting specimens for testing and delays in reporting of COVID-19 cases and deaths may impact the accuracy of the survival time estimates and the predicted trends for future deaths. Variations in guidelines that define COVID-19 cases and deaths, as well as framework conditions that prioritise certain subgroups for SARS-CoV-2 testing, make the comparison of fatality rates and survival times between different clinical settings and geographic regions challenging.

The predictive model presented in this study was based on a survival time of seven days. This allowed predictions to be extended to seven days from the end of the study period. Although additional modelling techniques would be required to extend estimates further into the future, such estimates would likely suffer from greater uncertainty over their accuracy. Although uncertainty is not eliminated completely from the estimates presented in this study, the short-term nature of the predictive model reduces these uncertainties to a minimum.

The seven-day survival time reported in this study was shorter than the 14-day survival time utilised by Baud et al. in their estimates of the global COVID-19 fatality rate that were calculated from data reported by the World Health Organization [4]. The survival time for COVID-19 cases from the Diamond Princess cruise ship was also reported to be longer (13 days) [11]. However, the survival time for patients in England with laboratory-confirmed COVID-19 who die from SARS-CoV-2 infection may be shorter than other estimates. This may be due to patients in the UK being tested for COVID-19 based on clinical need during the first wave of SARS-CoV-2 infection. That said, a higher COVID-19 fatality rate of 34% was reported for residents in a long-term community care facility in King County (Washington, USA) [22], suggesting that fatality rates among specific high-risk subgroups, such as frail, elderly, and immunosuppressed patients, may even be higher than the results presented in this study. Conversely, the fatality rates among children, in whom COVID-19 may be mild or asymptomatic, may be much lower, even in those requiring in-patient care.

## Conclusions

The model presented in this study provides a simple method for predicting laboratory-confirmed COVID-19 deaths. The fatality rate for patients in England with a clinical need for SARS-CoV-2 testing is 21.9%. These results, and the method upon which they are based, have utility for clinicians and policymakers for emergency preparedness and planning of clinical services. Precise measurements of the survival time of patients with the clinical need of COVID-19 testing may improve resource planning including the allocation of intensive care beds. Future research should (a) investigate the fatality rate and survival time of different subpopulations from different geographical subregions (including high-risk and ethnic minority groups) to aid our understanding of the natural history of COVID-19 and (b) should validate the current model with up-to-date data which includes cases and deaths from the second wave of SARS-CoV-2 infection.
